# “Self-cleaving” 2A peptide from porcine teschovirus-1 mediates cleavage of dual fluorescent proteins in transgenic *Eimeria tenella*

**DOI:** 10.1186/s13567-016-0351-z

**Published:** 2016-06-28

**Authors:** Xinming Tang, Xianyong Liu, Geru Tao, Mei Qin, Guangwen Yin, Jingxia Suo, Xun Suo

**Affiliations:** State Key Laboratory of Agrobiotechnology and College of Veterinary Medicine, China Agricultural University, Beijing, 100193 China; National Animal Protozoa Laboratory and College of Veterinary Medicine, China Agricultural University, Beijing, 100193 China; Key Laboratory of Animal Epidemiology and Zoonosis of Ministry of Agriculture, China Agricultural University, Beijing, 100193 China

## Abstract

The “self-cleaving” 2A sequence of picornavirus, which mediates ribosome-skipping events, enables the generation of two or more separate peptide products from one mRNA containing one or more “self-cleaving” 2A sequences. In this study, we introduced a single 2A sequence of porcine teschovirus-1 (P2A) linked to two fluorescent protein genes, the enhanced yellow fluorescent protein (EYFP) gene and the red fluorescent protein (RFP) gene, in a single cassette into transgenic *Eimeria tenella* (EtER). As expected, we obtained two separated protein molecules rather than a fused protein, although the two molecules were translated from the same mRNA carrying a single “self-cleaving” 2A sequence. Importantly, RFP led by a secretion signal was secreted into parasitophorous vacuoles, while EYFP localized mainly to the nucleus of EtER. Our results demonstrate that the “self-cleaving” 2A sequence actively mediated cleavage of polyproteins in the apicomplexan parasite *E. tenella.*

**Electronic supplementary material:**

The online version of this article (doi:10.1186/s13567-016-0351-z) contains supplementary material, which is available to authorized users.

## Introduction, methods and results

*Eimeriatenella* is an emerging model organism for studying the basic cell biology of protozoan parasites. Both transient and stable transfection systems in *Eimeria* species were developed successfully [1–4]. Transgenic parasites expressing fluorescent proteins, such as enhanced yellow fluorescent protein (EYFP), such as reporters, are particularly convenient to visually study the basic biology of *Eimeria* parasites [5–7]. The simultaneous expression of more than one reporter gene in a single construct is sometimes required, especially for studying multiple gene functions in different cellular compartments or organelles of organisms like the *Eimeria* parasite. So far, two open reading frames (ORF), each flanked with one set of regulatory sequences in a single plasmid (the so-called double cassette system) have been proven to be the only efficient way for co-expressing dual fluorescent protein genes in *Eimeria* parasites [4, 7, 8]. However, suppression of gene expression may occur in a single plasmid with multiple promoters due to the phenomenon called promoter interference [9].

This limitation has been overcome by a small “self-cleaving” peptide, F2A, first identified by Ryan et al. in the foot-and-mouth disease virus, a member of the picornavirus group [10]. Subsequently, “2A like” peptides from equine rhinitis A virus (E2A), porcine teschovirus-1 (P2A) and *Thosea asigna* virus (T2A) were identified, and their activities in proteolytic cleavage were shown in various in vitro and in vivo eukaryotic systems [10–13]. Here, we report a single cassette system which co-expresses two fluorescent proteins, EYFP and RFP (red fluorescent protein), and is cleaved by P2A in the apicomplexan parasite *E. tenella*.

**Figure 1:**
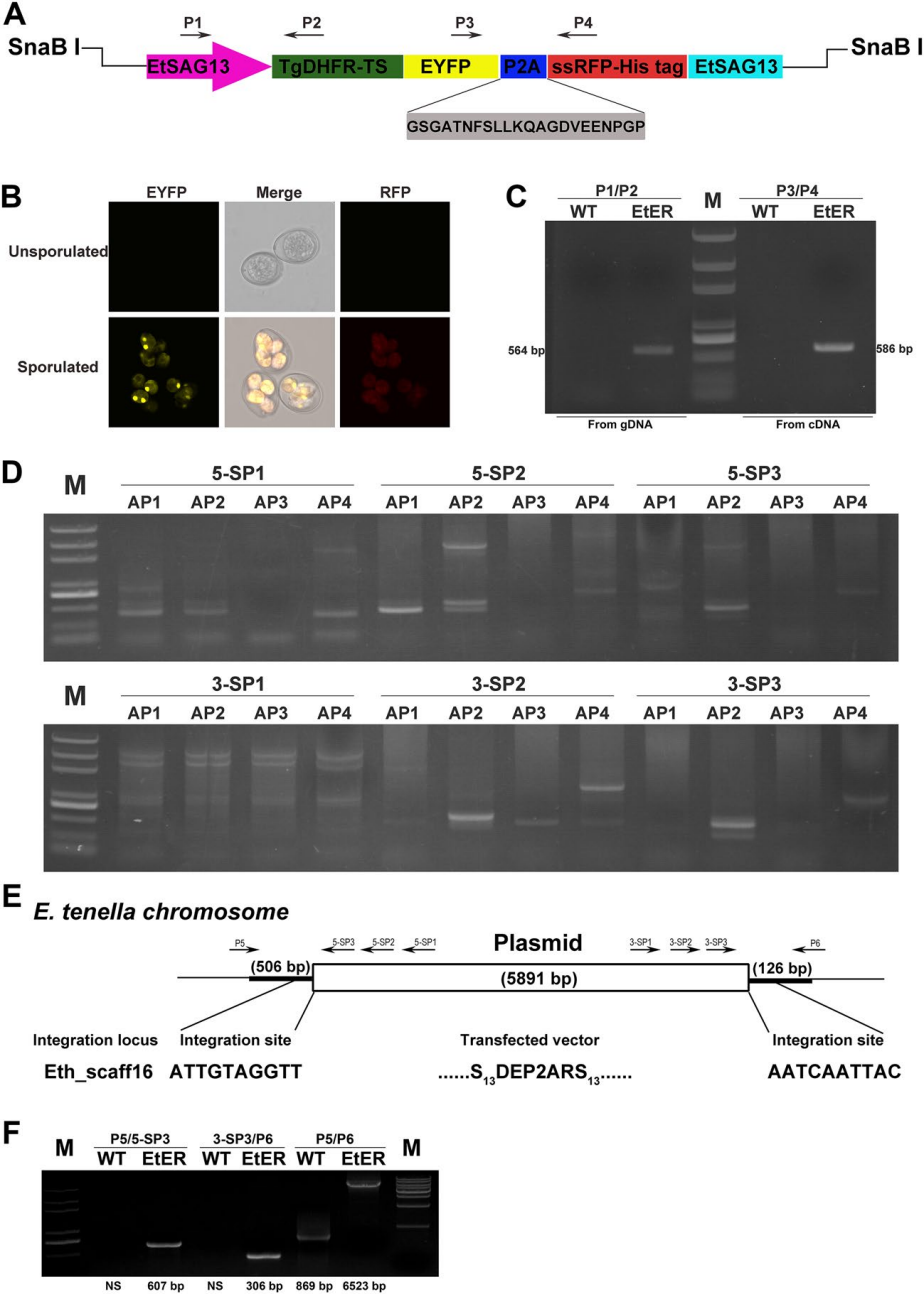
**Generation of stably transfected** ***E. tenella*** **line co-expressing EYFP and RFP.** **A** Schematic of the transfected vector (pSDEP2ARS). 5′ UTR (1376 bp) and 3′ UTR (1002 bp) of *E. tenella* surface antigen 13 (SAG13) were amplified from genomic DNA with primers SAG13-5-F/SAG13-5-R and SAG13-3-F/SAG13-3-R (Additional file 1), respectively. The synthesized 66 bp nucleotides encoding porcine teschovirus 2A sequence (Gray background and Additional file 1) was fused with the RFP gene of pMIC-EYFP/ACTss-RFP [7] by three rounds of PCR (2A-F1/2A-F2/2A-F3 and 2A-R (Additional file 1). ss: *T. gondii* GRA8 signal sequence. **B** Both EYFP and RFP were expressed in sporulated oocysts whereas no fluorescence could be detected in unsporulated oocysts. **C** Genomic DNA and cDNA from EtER was amplified with the primers P1/P2 (giving a 564 bp product) or P3/P4 (giving a 586 bp product) to verify the recombination of EYFP and RFP genomic DNA and cDNA from wild type *E. tenella* were used as a control. M: marker. **D** Genomic DNA from EtER was amplified with arbitrary degenerate primers (AP 1, AP 2, AP 3 and AP 4) and specific primers (5-SP 1, 5-SP 2 and 5-SP 3/3-SP1, 3-SP2 and 3-SP3 (Additional file 1) from SAG13 5′ (upper) and 3′ (lower) UTR by thermal asymmetric interlaced PCR, and the products from the third-round PCR were cloned into pEasy-T1 vector for sequencing. M: marker. **E** One integration site (Eth_scaff16) was confirmed by BLAST from 58 clones in *E. tenella* GeneDB. **F** Genomic DNA from EtER was amplified with the primers P5/5-SP3 (giving a 607 bp product), 3-SP3/P6 (giving a 306 bp product) and P5/P6 (giving a 6523 bp product) to verify the integration site of transfected vector in Eth_scaff16 locus in the EtER genome. Genomic DNA from wild type *E. tenella* was used as a control. M: marker, NS: no specific band.

Surface antigen 13 (SAG13) is a highly expressed sporozoite antigen of *E. tenella*. The expression level of SAG13 is nearly 5% of *E. tenella* total sporozoite soluble antigens [14]. We constructed a single cassette system in which TgDHFR/EYFP-P2A-ssRFP was flanked by the SAG13 promoter and its 3′ UTR (Figure 1A; Additional file 1). TgDHFR (pyrimethamine resistance gene) was used for transgenic parasite selection. P2A was a 66-base pair oligonucleotide from porcine teschovirus-1 [15]. RFP was preceded by GRA8, a signal sequence, and followed by a His6 tag [7]. Restriction enzyme-mediated integration (REMI) was adapted for the transfection of sporozoites as previously described [16, 17]. Five 3-day-old AA broilers were inoculated with 10^6^ electroporated sporozoites via the cloacal route. Chicks were given a standard diet supplemented with 150 ppm pyrimethamine (Sigma, USA) 18 h after inoculation [18]. Oocysts from faeces excreted 6–10 days post-inoculation (dpi) were collected and sporulated for an additional four generations of in vivo passages as previously described [19].

We then infected 30 two-day-old AA broilers with single transgenic parasite oocysts from the fifth generation and obtained a stable transgenic line, EtER (Additional file 2) [2, 20]. The stable line expressed EYFP and RFP in its sporulated oocyst stage but not in its unsporulated oocysts (Figure 1B), showing that the SAG13 promoter is a stage-specific regulatory sequence [21].

To further characterize the transgenic strain at the molecular level, we extracted DNA and RNA from EtER as previously described [20, 22]. cDNA was synthesized from the extracted RNA using random primers and a high capacity cDNA Reverse Transcription Kit (Applied Biosystems) [22]. PCR-based analysis using P1/P2 primers revealed that the transfection vector existed in the transgenic parasite genome (gDNA), and analysis using P3/P4 primers revealed that EYFP-RFP was translated by the same mRNA from cDNA (Figures 1A and C). We further analysed the integration sites of the 5′ and 3′ ends of the transfected vector in the EtER genome through genome walking (Figures 1D and E) [6, 17]. Specific primers (5-SP 1, 5-SP 2, 5-SP 3/3-SP1, 3-SP2 and 3-SP3; Additional file 1) were designed according to the SAG13 promoter and 3′ UTR sequence following the kit instructions. The flanking sequences to the 5′ and 3′ ends of the integrated vector were obtained after three rounds of thermal asymmetric interlaced PCR. We found that both the 5′ and 3′ ends of the exogenous plasmid were integrated in the Eth_scaff 16 locus (Figure 1E). To confirm that the exogenous vector was integrated into that specific site of the parasite genome, we designed specific primers, P5 and P6 (Figure 1E; Additional file 1), based on the 5′ and 3′ flanking sequences of the transfected vector in the Eth_scaff 16 locus. After amplification with various primer pairs (P5/5-SP3, P6/3-SP3 and P5/P6, Figure 1F; Additional file 1) from wild type *E. tenella* and EtER genomic DNA, we obtained the predicted specific bands from EtER genomic DNA (Figure 1F). All these data show that we obtained a stable transgenic *E. tenella* line (EtER).

**Figure 2:**
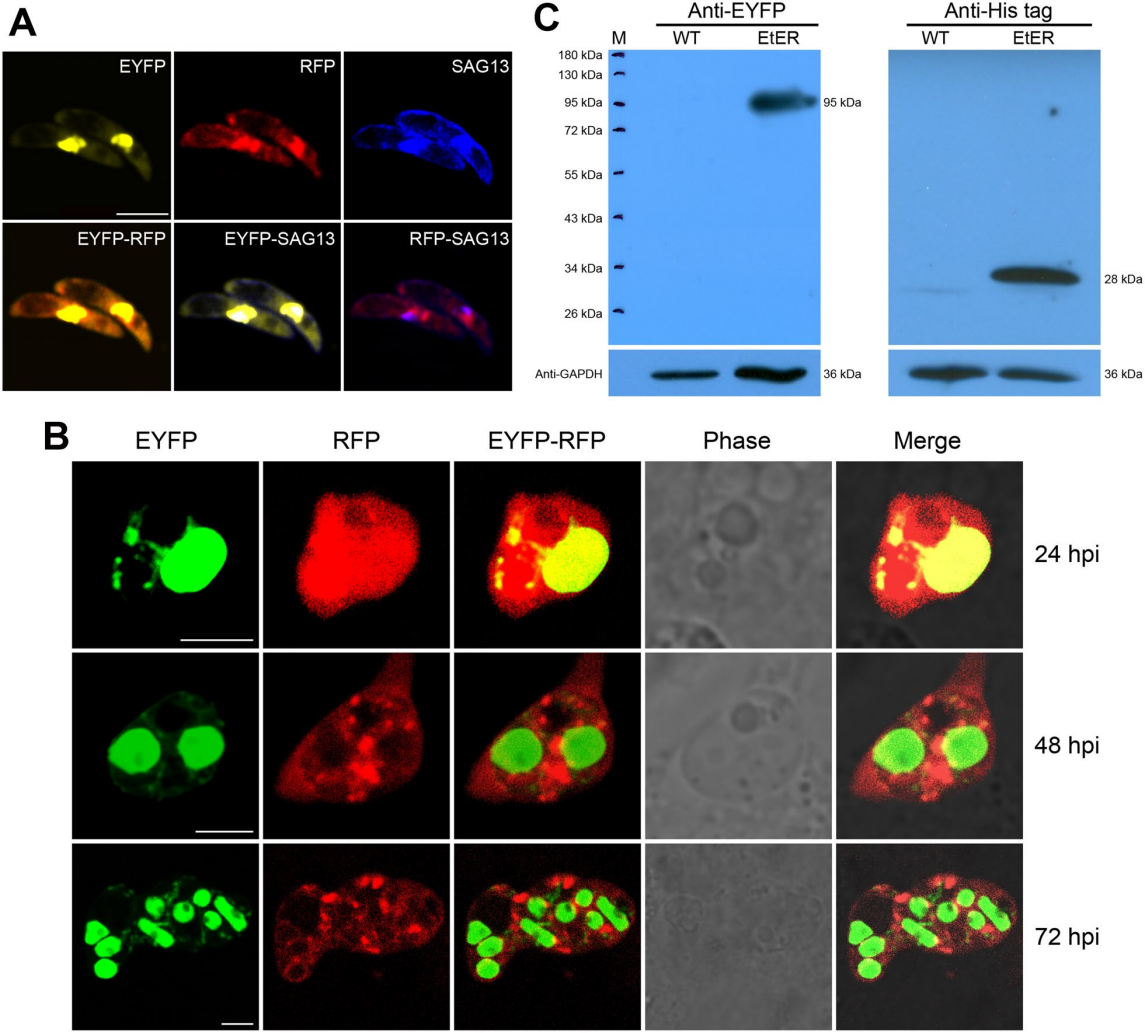
**“Self-cleaving” 2A peptide cleaves EYFP and RFP in EtER.** **A** EtER sporozoites were reacted with mouse anti-SAG13 polyclonal antibody, followed by the reaction with AMCA-conjugated goat anti-mouse IgG (H + L) (Proteintech, USA), revealed by a confocal laser scanning microscopy (SP5, Leica, Germany). EYFP mainly localized to the nucleus, while RFP was found in the cytoplasm. Bar 5 μm. **B** RFP was secreted into PV in trophozoites (24 hpi) and 1^st^-genernation schizont stages, while EYFP in the nucleus (48 and 72 hpi). Bar 5 μm. **C** Soluble proteins extracted from EtER and the wild-type *E. tenella* (WT) were resolved by SDS-PAGE and the immunoblot analysis was conducted following standard protocols. The primary antibody was the mouse anti-EYFP polyclonal antibody and mouse anti-His tag monoclonal antibody, the mouse anti-GAPDH polyclonal antibody served as the loading control, while the HRP-conjugated goat anti-mouse IgG was used as the secondary antibody. TgDHFR-EYFP was 95 kDa and RFP 28 kDa. M: marker.

To assess if the “self-cleaving” 2A peptide could mediate proteolytic cleavage in transgenic *E. tenella* and to show that the introduction of a signal peptide to regulate RFP secretion did not interfere with EYFP and RFP distribution in EtER, (Figure 1A) [7, 23], we conducted an indirect immunofluorescence assay (IFA). In this assay, EtER sporozoites were treated with mouse anti-SAG13 polyclonal-antibody, followed by AMCA-conjugated goat anti-mouse IgG staining for confocal microscope imaging. We found that EYFP mainly localized to the nucleus, while RFP was found in the cytoplasm (Figure 2A). We also found that SAG13 was distributed in the cell surface, with some observed nuclear SAG signal interpreted as bleeding of EYFP (Figure 2A).

To confirm that the function of the GRA8 signal peptide was not affected by P2A, we did an in vitro culture of primary chicken kidney cells (PCKC) inoculated with EtER sporozoites. As expected, EYFP localized mainly to the nucleus, while RFP was secreted into parasitophorous vacuoles (PV) in trophozoites [24 h post-inoculation (hpi)] and 1st-generation schizont stages (48 and 72 hpi) (Figure 2B) [24]. As a stronger promoter was used here, there are many red signals in the cytoplasm (Figure 2B).

To further test the self-cleavage efficiency of P2A in EtER, we used Western blotting. EtER sporozoite soluble antigens were subjected to SDS-PAGE, followed by reactions with mouse anti-EYFP polyclonal-antibody, mouse anti-His tag monoclonal antibody and HRP-conjugated goat anti-mouse IgG (Proteintech, USA) secondary antibody. Our results showed that aTgDHFR-EYFP (95 kDa) was efficiently cleaved from RFP (28 kDa) (Figure 2C).

The above results conclusively demonstrate that the “self-cleaving” 2A sequence of picornavirus works efficiently in the apicomplexan parasite *E. tenella.*

## Discussion

Expression of multiple polypeptides from a single mRNA is made possible by inclusion of a short viral 2A peptide coding sequence between the polypeptide-encoding transgenes [11, 25]. In this study, we obtained a stably transfected *E. tenella* line (EtER) expressing separate EYFP and RFP from one mRNA mediated by P2A function. Our result encourages the use of P2A in cell biological studies of apicomplexan parasites that may require multi-reporter expression in a single cassette.

Interestingly, we found that SAG13 was a much stronger promoter than His4 as revealed by EYFP density via the confocal imaging measurement (data not shown), which indicates the usefulness of SAG13 for the study of gene overexpression without modifying the genome [26]. However, overexpression may also result in the appearance of unusual biological characteristics in transgenic parasites compared to wild-types [2].

Our previous study showed that transgenic *E. tenella* expressing EYFP in different cellular compartments elicit EYFP-specific, systemic and mucosal immune responses [22], and another group showed that *E. tenella*-delivered CjA stimulates protective immunity against *Campylobacter jejuni* infections in chickens [27]. The finding that P2A mediates separate expression of multiple genes in different compartments has great implications for the development of transgenic *Eimeria* parasites as vaccine vectors and beyond [2, 6, 7, 16, 17, 20, 22].

## Additional files

10.1186/s13567-016-0351-z **Primers used in this study.** Primers and P2A sequences were listed in this file.
10.1186/s13567-016-0351-z **Transgenic parasite (EtER) selection.** The details of EtER selection including the inoculation dosage of each generation and the efficacy of drug selection are provided.

## Abbreviations

P2A: 2A sequences from porcine teschovirus-1
EYFP: enhanced yellow fluorescent protein
RFP: red fluorescent protein
EtER: transgenic *Eimeria tenella* line co-expressing EYFP and RFP
SAG13: *Eimeria tenella* sporozoites surface antigen 13
GRA 8: *Toxoplasma gondii* dense granule 8
