# Supplementary material for: “Self-cleaving” 2A peptide from porcine teschovirus-1 mediates cleavage of dual fluorescent proteins in transgenic Eimeria tenella
Source: Vet Res. 2016 Jun 28;47:68. doi: 10.1186/s13567-016-0351-z (PMC4924277; doi:10.1186/s13567-016-0351-z)
Supplement: Supplementary file 1 — 10.1186/s13567-016-0351-z Primers used in this study. Primers and P2A sequences were listed in this file. [file 13567_2016_351_MOESM1_ESM.docx]

**Additional file 1** **Primers used in this study.**

| Primer name | Primer sequence (5’ to 3’) | Notice |
| --- | --- | --- |
| SAG13-5-F  SAG13-5-R  SAG13-3-F  SAG13-3-R  2A-F1  2A-F2  2A-F3  2A-R  P1  P2  P3  P4  5-SP 1  5-SP 2  5-SP 3  3-SP 1  3-SP 2  3-SP 3  P5  P6 | TACGTACCTAGGCGGGAAAGGCACCTATGCTGCAA  GCGGCCGCTGCGGAAAAACACAGAAAGCAAA  CCGCGGATTTCGCGAAGGGCGTCAAACAAAGT  TACGTAGCTAGGGAATGATGCGTTTTCACCAAC  GAGGAGAACCCTGGACCTCATATG  ATGGCTTTACCATTGCGTGT  **AACTTCAGCCTGCTGAAGCAGGCTGGAG**  **ACGTGGAGGAGAACCCTGGACCT**  GGTACC**GGAAGCGGAGCTACT**AACTTCA  GCCTGCTGAAGCAG  CCGCGGTCA*GTGGTGGTGGTGGTGGTG*  CTGGGAGCCGGAGTGGC  AGTCACTAGTGGAATAACCGCGCA  GCTTTCCCAGTTTTTCCGTCC  AACCACTACCTGAGCTACCAGTC  TGGAGCGCTCCCAGGTGAA  GTCTCTGACCGCATCTCCCTCTAT  CCTATGATGCGTGTAAGTGGTGGAC  CTACTGGTACATGCGCAAGCGTCA  CACAAGCTACTTGGCACCCATGTG  ACACTCCTCGTGCAGACGAATAGC  GAGCACGCACACACAGTCATAGT  TAAGCAAGTAGCTGCCCGCCT  GCGAACCTAGCAAGATGTCACG | Avr II  Not I  Sac II  SnaB I  Nde I  **P2A**  Kpn I  Sac II  *His tag* |
