# Supplementary material for: “Self-cleaving” 2A peptide from porcine teschovirus-1 mediates cleavage of dual fluorescent proteins in transgenic Eimeria tenella
Source: Vet Res. 2016 Jun 28;47:68. doi: 10.1186/s13567-016-0351-z (PMC4924277; doi:10.1186/s13567-016-0351-z)
Supplement: Supplementary file 2 — 10.1186/s13567-016-0351-z Transgenic parasite (EtER) selection. The details of EtER selection including the inoculation dosage of each generation and the efficacy of drug selection are provided. [file 13567_2016_351_MOESM2_ESM.docx]

**Additional file 2 Transgenic parasite (EtER) selection**.

| **Reporter** | **Generation** | **Inoculate dosage (Oocysts/bird)** | **% Parasite expressing** | **Oocysts output/bird** | **Next selection** |
| --- | --- | --- | --- | --- | --- |
| EYFP | 1  2  3  4  5  6  7 | 1 × 10^6^ (sporozoites)  1000  5000  5000  5000  1 (30)  100 | 12.3  57.3  78.5  82.3  89.2  100 (1/30)  100 | 1.2 × 10^4^  2.3 × 10^7^  5.1 × 10^7^  4.8 × 10^7^  2.8 × 10^8^  ~300  1.4 × 10^6^ | Drug+FACS  Drug  Drug  Dug  ---  ---  --- |
